# Supplementary figures and images for: Efficacy of surgical management for recurrent intrahepatic cholangiocarcinoma: A multi-institutional study by the Okayama Study Group of HBP surgery
Source: PLoS One. 2020 Sep 3;15(9):e0238392. doi: 10.1371/journal.pone.0238392 (PMC7470360; doi:10.1371/journal.pone.0238392)

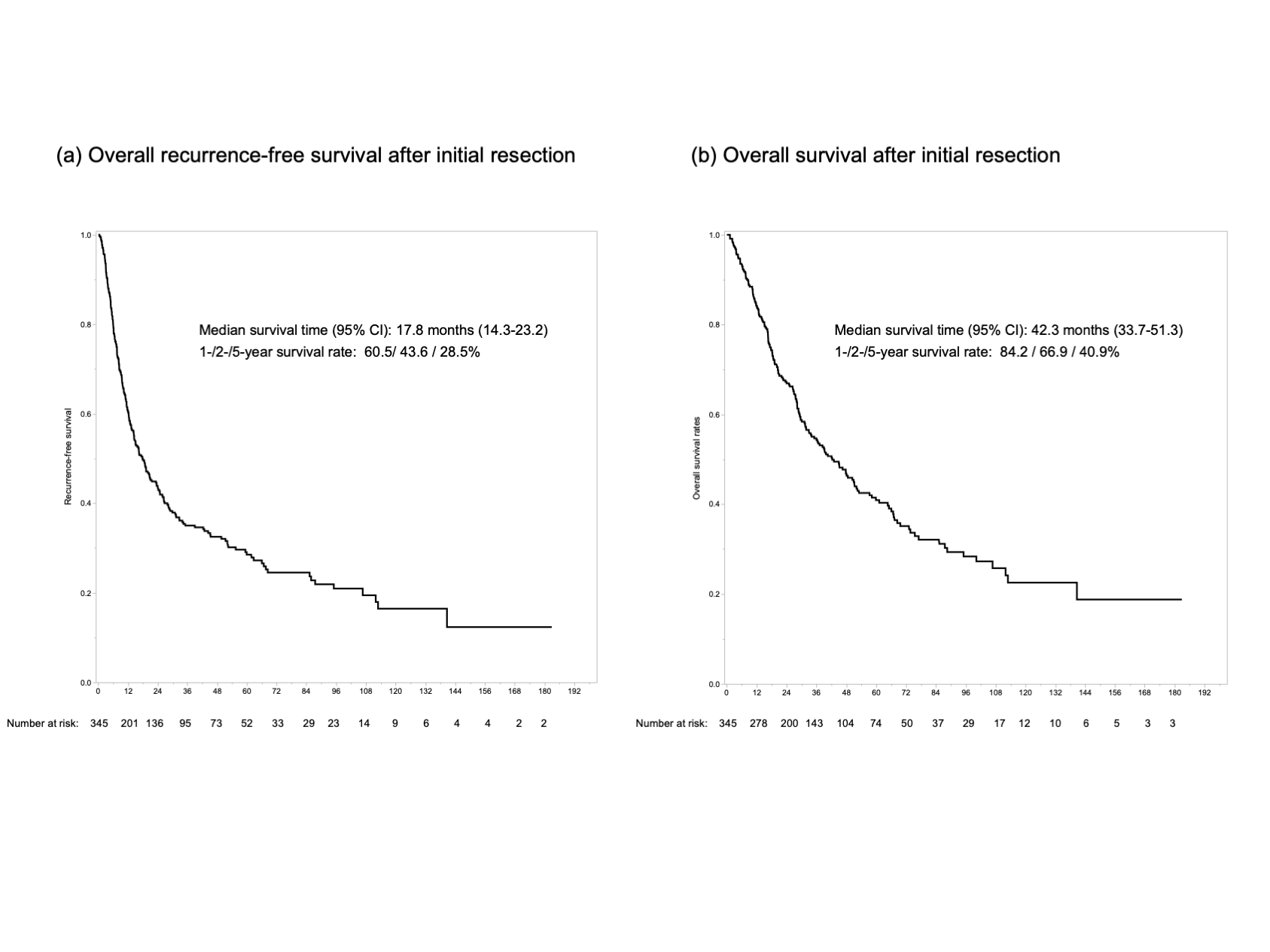

Supplement: S1 Fig — (TIFF) [file pone.0238392.s001.tiff]
