# Supplementary material for: Efficacy of surgical management for recurrent intrahepatic cholangiocarcinoma: A multi-institutional study by the Okayama Study Group of HBP surgery
Source: PLoS One. 2020 Sep 3;15(9):e0238392. doi: 10.1371/journal.pone.0238392 (PMC7470360; doi:10.1371/journal.pone.0238392)
Supplement: Attachment — Submitted filename: PlosOne Response to Reviewers.doc [file pone.0238392.s002.doc]

Aug 3, 2020

Gianfranco D. Alpini
Academic Editor
PLOS ONE

Manuscript ID: PONE-D-20-19960

Title: Efficacy of surgical management for recurrent intrahepatic cholangiocarcinoma: A multi-institutional study by the Okayama study group of HBP surgery

Authors: Kojima T et al.

Dear Dr. Alpini:

Thank you for your e-mail of July 17, 2020. We were pleased to know of your positive evaluation of our manuscript and its potential acceptance for publication in PLOS ONE, subject to adequate revision and response to the reviewers' comments.

Based on your instructions, we uploaded the clean and marked files of the revised manuscript. We also pasted in the allocated space on the website our point-by-point responses to the comments raised by the reviewer.

To allow easy access to our response to the comments raised by the reviewer, we also include a copy of our response in this letter. Essentially, we agreed with all the comments raised by the reviewer.

We take this opportunity to thank the reviewer for the hard work. We also thank you for allowing us to resubmit a revised copy of the manuscript.

I hope that the revised manuscript is now acceptable for publication in PLOS ONE.

Sincerely Yours,

Yuzo UMEDA, MD, PhD

Okayama University Graduate School of Medicine and Dentistry

Department of Gastroenterological Surgery

Shikata-cho, Okayama-shi, Okayama 700-8558, JAPAN

E-mail: [y.umeda@d9.dion.ne.jp](mailto:y.umeda@d9.dion.ne.jp)

=================================

Point-by-point response to the comments of Reviewer 1

We thank the reviewer for evaluating our manuscript. The following text describes our responses to the comments made by the reviewer.

1. The reviewer recommended a comprehensive statement about survival and recurrence rate in the Abstract. Accordingly, we changed the phrase “post-recurrence survival rates” to “survival rates after recurrence”.

Page 3, line 10 in the Abstract section.

2. We apologize for our inadequate table content. We mounted the proper formatted table 1, 2, and 3.

Page 8, Table 1 was inserted as the proper format.

Page 10-11, Table 2 was inserted as the proper format.

Page 12-13, Table 3 was inserted as the proper format.

=================================

Point-by-point response to the comments of Reviewer 2

We thank the reviewer for evaluating our manuscript. The following text describes our responses to the comments made by the reviewer.

1. We apologize for our inadequate table contents.

We modified all tables and submitted the proper formatted table 1, 2, and 3 in the revised document.

Page 8, Table 1 was inserted as the proper format.

Page 10-11, Table 2 was inserted as the proper format.

Page 12-13, Table 3 was inserted as the proper format.

=================================

Point-by-point response to the comments of Reviewer 3

We thank the reviewer for evaluating our manuscript and greatly appreciated the reviewer's very important and worthwhile comments. The following text describes our responses to the comments made by the reviewer. The line number mentioned in the response to the comment refers to the small-size number that appears on the left margin of the text of the revised manuscript.

1. As noted by the reviewer, patients undergoing surgery for recurrence had a lower rate of Hilar type ICC and did not undergo major hepatectomy compared to non-resected recurrent patients, and this may have influenced the underlying remnant liver function in their decision-making. In results and discussion, these aspects were added.

Page 15, line 8-10 in the discussion section.

2. We appreciate the valuable comments, as the Reviewer pointed out ‘the definition of hilar/peripheral ICC’ is a very important issue. Firstly, the tumor involving hilar bile duct were not included. And classification of hilar/peripheral type was practically based on preoperative imaging. So we provide the accurate definition of hilar/peripheral type ICC in this study.

Page 5, line 20 to Page 6 line 2 in the materials and methods section.

3. We agree with the prognostic impact of histomorphological subtypes. Unfortunately, due to the limitation of multicenter retrospective study, it was not possible to identify these subtypes in all resected cases.

Therefore, we added previous articles reporting on the correlation between tumor localization and histomorphology as a reference. The recurrence rate of the large bile type, represented by the Hilar type, was higher than that of the small bile type, represented by the peripheral type (n=94 [75.2%] vs n=129 [58.6%], p=0.0023).

We added reference #31-34 as a new reference.

Page 15, line 3-11 in the discussion section.
